# Lowered dietary phosphorus affects intestinal and renal gene expression to maintain mineral homeostasis with immunomodulatory implications in weaned piglets

**DOI:** 10.1186/s12864-018-4584-2

**Published:** 2018-03-20

**Authors:** Franziska Just, Michael Oster, Kirsten Büsing, Luisa Borgelt, Eduard Murani, Siriluck Ponsuksili, Petra Wolf, Klaus Wimmers

**Affiliations:** 1 Leibniz Institute for Farm Animal Biology (FBN), Institute for Genome Biology, Wilhelm-Stahl-Allee 2, 18196 Dummerstorf, Germany; 2 Faculty of Agricultural and Environmental Sciences, University Rostock, 18059 Rostock, Germany

**Keywords:** Calcium: Phosphorus ratio, Pig, Gene expression, Immune response, Diet

## Abstract

**Background:**

In monogastric animals, phosphorus (P) homeostasis is maintained by regulating intestinal absorption, bone mobilization, and renal excretion. Since P is a non-renewable resource, a shortage is imminent due to widespread over-usage in the farming and animal husbandry industries. As a consequence, P efficiency should be improved in pig production. We sought to characterize the transcriptional response in re−/absorbing and excreting tissues in pigs to diets varying in calcium: phosphorus ratios. Weaned piglets were assigned to one of three groups fed diets varying in digestible P content for a period of five weeks. Gene expression profiles were analyzed in jejunum, colon, and kidney.

**Results:**

Transcriptome analysis revealed that reduced dietary P intake affects gene expression in jejunum and kidney, but not in colon. The regulation of mineral homeostasis was reflected via altered mRNA abundances of *CYP24A1*, *CYP27A1*, *TRPM6*, *SPP1*, and *VDR* in jejunum and kidney. Moreover, lowered abundances of transcripts associated with the classical complement system pathway were observed in the jejunum. In kidney, shifted transcripts were involved in phospholipase C, calcium signaling, and NFAT signaling, which may have immunomodulatory implications.

**Conclusions:**

Our results revealed local transcriptional consequences of variable P intake in intestinal and renal tissues. The adaptive responses are the result of organismal efforts to maintain systemic mineral homeostasis while modulating immune features at local tissue sites. Therefore, the deviation from the currently recommended dietary P supply must be carefully considered, as the endogenous mechanisms that respond to low P diets may impact important adaptive immune responses.

**Electronic supplementary material:**

The online version of this article (10.1186/s12864-018-4584-2) contains supplementary material, which is available to authorized users.

## Background

Over the last few decades, efforts to conserve the non-renewable resource phosphorus (P) have become increasingly prominent in scientific research. In mammals, the biologically active form, phosphate $\left({\mathrm{PO}}_{4,}^{3-}\right)$, is an essential structural component of nucleic acids, phospholipids, adenosine triphosphate (ATP), and hydroxyapatite in bone. P homeostasis is controlled by absorption in the small intestine (especially in the jejunum), bone remodeling, and reabsorption/excretion in the kidney [1]. In farmed pigs, a deficient P intake could lead to less bone formation and, therefore, may reduce weight gain [2, 3]. To avoid P deficiency symptoms and to ensure maximal growth, dietary P is often supplemented to levels exceeding age-specific requirements, with up to two-thirds of the consumed P being excreted [4, 5]. In agriculture, the majority of P input is the result of mineral fertilizers and animal manure, which are used to encourage crop production [6]. Unfortunately, over-application and accumulation of P results in P-laden runoff, leading to pollution and eutrophication of surface waters [7, 8].

To deal with the environmental concerns surrounding P usage, increased efficiency should be considered in pig husbandry. P recommendations should be re-evaluated considering the requirements of modern pig breeds and the mechanisms of P utilization, which are known to be affected by genetic factors and transcriptional regulation [9]. To date, only a few studies have described the molecular mechanisms that respond to varying P supplementation in pigs. Activation of vitamin D and increased P uptake from the intestine are widely accepted. Specifically, a low P diet prompted an increased mRNA abundance of 1α-hydroxylase and parathyroid hormone receptor [10], and an increased sodium-dependent phosphate uptake in the jejunum in pigs [11]. Furthermore, a previous study of our group investigated the transcriptional response of peripheral blood mononuclear cells to diets varying in calcium (Ca):P ratios, showing P as a link between bone remodeling and immune features [12]. In fact, an impact of P on the immune system has also been observed in other farm animals where e.g. dietary effects on T cell function, lymphocyte proliferation and antibody production have been reported [13–15].

The intestinal mucosa is of crucial importance to immune regulation due to its continuous contact with the external environment. In particular, the gut-associated lymphoid tissue (GALT), including Peyer’s patches, mediates the homeostatic balance between tolerance of commensal microorganisms and immune response to pathogens [16, 17]. Moreover, the mucosa-associated bacterial community can be influenced by dietary compositions [18]. The kidney also has a special role in immune tolerance against dietary antigens and hormones [19]. Furthermore, the kidney contributes to the production of hormones with immunomodulatory properties, including vitamin D [20]. Hence, several local immunological processes are present in different tissues and these may be orchestrated by systemic endocrine processes.

To evaluate the consequences of reducing dietary P supplementation in swine production, we sought to characterize the organismal response to variable P supply in pigs. The endocrine regulation of P homeostasis due to diets varying in Ca:P ratios has been previously reported [12]. Here, we describe the transcriptional effects of dietary P on tissues that are involved in P re−/absorption and excretion such as jejunum, colon, and kidney.

## Methods

### Animals and diets

Animals were provided by the Leibniz Institute for Farm Animal Biology (FBN). The experimental protocol was approved by the ethics committee of the federal state of Mecklenburg-Western Pomerania, Germany (Landesamt für Landwirtschaft, Lebensmittelsicherheit und Fischerei; LALLF MV 7221.3–1-053/15). The experimental design was described previously [12]. In short, 19 German landrace piglets from three litters of two boars were randomly assigned to one of three dietary groups (selection according to gender and litter balanced). For a period of 5 weeks (day 28–64), piglets were fed diets which differed in relation to the digestible P-content (Additional file 1). Neither phytase nor other phosphatases were added. Specifically, the achieved levels of digestible P were 0.33% (low; L), 0.51% (medium; M) and 0.74% P (high; H) on a dry matter basis. The dietary P content in group M corresponded to current recommendations [21].

### Collection and preparation of tissue samples

At 64 days of age, pigs were killed by electrical stunning followed by exsanguination in the experimental slaughterhouse of FBN. Kidney, jejunum, and colon tissue were immediately collected, frozen in liquid nitrogen, and stored at − 80 °C.

### RNA isolation, target preparation, and hybridization

Total RNA was isolated using TRI Reagent per manufacturer’s directions (Sigma-Aldrich, Taufkirchen, Germany), then treated with DNase and purified with the column-based NucleoSpin RNA II-Kit (Macherey-Nagel, Düren, Germany). RNA integrity was determined by visualization on a 1% agarose gel containing ethidium bromide and the concentration was measured using the NanoDrop ND-1000 spectrometer (PEQLAB, Erlangen, Germany). DNA contamination was assessed by PCR amplification of the porcine RPL32 gene (forward primer: 5’-AGCCCAAGATCGTCAAAAAG-3′; reverse primer: 5’-TGTTGCTCCCATAACCAATG-3′). All RNA samples were stored at − 80 °C.

Each RNA sample was transcribed to DNA using the Ambion WT Expression Kit (Ambion, Austin, TX, USA). The DNA preparations were fragmented and labelled with the WT Terminal Labeling Kit (Affymetrix, Santa Clara, CA, USA). DNA preparations were hybridized on genome-wide *snowball* arrays (Affymetrix), which were invented for genome-wide analysis of the pig transcriptome [22]. Raw data was generated with Affymetrix GCOS 1.1.1 software and deposited in a MIAME-compliant database [23], the National Center for Biotechnology Information Gene Expression Omnibus (www.ncbi.nlm.nih.gov/geo; accession number: GSE94448).

### Microarray data processing and analysis

All arrays were tested and passed quality control criteria as proposed previously [24]. The data were RMA (Robust Multiarray Average) [25] normalized (Log2) and filtered by both standard deviation (SD > 0.29) and mean (x̅> 2.5) using R version 3.2.0 [26]. Transcriptional effects were analyzed between samples from animals fed L, M, and H diets. Relative changes of transcript abundance were analyzed using a linear model (JMP Genomics 7.0), including the effects of diet, sire, sex, tissue, and interactions (V_ijkl_ = μ + diet_i_ + sire_j_ + sex_k_ + tissue_l_ + diet_i_*tissue_l_ + error_ijkl_). *P*-values were converted to a set of q-values to correct for multiple testing using the algorithm proposed by Storey & Tibshirani [27]. Significance levels were set at *p* ≤ 0.05 and q ≤ 0.20. The annotation data for *snowball* arrays were obtained from the developers [22]. Analysis of regulated pathways was performed using the web-based software Ingenuity Pathway Analysis (IPA) version 01–04 (Qiagen, Redwood City, CA, USA). The significance of association between altered transcripts and regulated pathways was set at *p* ≤ 0.05. Genes with shifted transcript abundance were used to identify the molecular and cellular functions affected by P supply using IPA ‘Diseases and Bio-functions’ (criteria: z-score ≥ 2 or z-score ≤ − 2). To visualize the differences in the gene expression of the investigated tissues, a non-parametric hierarchical cluster analysis of significantly different probe-sets was performed using R (*hclust* option ‘complete’; *dist* option ‘euclidean’). In total 29,013 probe-sets were used to generate the heatmap.

### Quantitative real-time RT-PCR

All RNA samples were transcribed to cDNA. First-strand cDNA was synthesized from 2 μg of total RNA using random hexamer primers and oligo d(T)_13_VN in the presence of SuperScript III reverse transcriptase (Invitrogen, Karlsruhe, Germany). To verify the microarray experiments, total transcript levels of selected targets and reference genes (Additional file 2) were quantified by real-time quantitative PCR (qPCR) performed on a LightCycler®480 system (Roche, Mannheim, Germany). Target genes involved in observed immunoregulatory pathways (*NFATC2*, *SPP1*, *C1QC*, *C7*, *C1R*, *C1S*, and *MMP2*) and in P metabolism (*CYP24A1, CYP27A1*, *PTH1R*, *SLC34A3* and *VDR*) were selected for analysis. Reactions were accomplished in a final volume of 12 μL using 6.0 μL of LightCycler 480 SYBR Green I Master (Roche, Basel, Switzerland), 2.8 μL of *Aqua dest*., 0.6 μL of each Primer (10 μM) and 2 μL (40 ng) cDNA. Amplifications were conducted in duplicate according to the manufacturer’s instructions. The temperature profiles of PCR were as follows: initial denaturation step at 95 °C for 10 min and 40 cycles including denaturation at 95 °C for 15 s, annealing at 60 °C for 10 s and extension/fluorescence acquisition at 72 °C for 15 s. After completion of amplification, melting curve analysis and agarose gel electrophoresis were conducted to confirm the absence of unspecific PCR products or primer dimers. Serial dilutions of external PCR-generated standards (10^8^–10^2^ copies) were used to convert the threshold cycles into copy numbers. The calculated copy numbers were factorial normalized on *RPL32* expression values. Data were calculated using a linear model analysis (JMP Genomics 7.0; effects of diet, sire, and sex). Significance levels were set at *p* ≤ 0.05. The analyzed data was compared to microarray results using the Spearman’s Rank Correlation in R version 3.2.0.

## Results

To characterize the effects of modulated dietary phosphorus supply, we analyzed the transcriptomic profiles of tissues responsible for re−/absorption (jejunum and colon) and excretion (kidney).

### Transcriptome profiles

The *snowball* microarray covers 47,845 probe-sets corresponding to 17,964 annotated genes. After filtering as described above, 29,013 probe-sets (~ 61%) remained for analysis. These probe-sets corresponded to 14,064 annotated genes. The number and annotation of regulated transcripts (per diet and tissue) is shown in Additional file 3.

In jejunum, two probe-sets differed between groups M and H (2 probe-sets M > H). The comparison between groups L and H showed changed mRNA abundance of 168 probe-sets (72 probe-sets L > H). Between groups L and M, 172 probe-sets (129 probe-sets L < M) differed significantly. Of these, 48 probe-sets (41 probe-sets L < M and L < H) were differentially expressed in both comparisons, i.e. L vs. H and L vs. M.

Diet-dependent gene expression patterns in colon showed only marginal transcriptional alterations. Between groups H and M, seven probe-sets (5 probe-sets H > M) differed significantly. In group L, two probe-sets were increased compared to group M. There were no pathways found to be enriched.

In kidney, 74 probe-sets (20 probe-sets L > H) were altered between groups L and H. The comparison between groups L and M revealed 330 differentially expressed probe-sets (167 probe-sets L > M). Of these, 47 probe-sets (38 probe-sets L < M and L < H) were differentially expressed in both comparisons.

Differences in gene expression were visualized by hierarchical clustering (Additional file 4). The heat map shows three major clusters representing the different tissues, whereby the colon and jejunum showed less distance due to gene expression patterns. The kidney was separated from the intestinal tissues. Group L showed greater distance from groups M and H in kidney and jejunum, reflecting the outcome of the parametric statistical analyses.

### Transcriptional regulation of signaling pathways and bio-functions

IPA analysis revealed a number of P-regulated canonical pathways and Bio-functions in jejunum and kidney. In colon, there were no pathways and Bio-functions enriched.

In the jejunum, gene expression pattern revealed the same top three regulated pathways for the comparisons L vs M and L vs H (Table 1). ‘Hepatic Fibrosis’, ‘RhoA Signaling’, and ‘VEGF Signaling’ are associated with a number of genes encoding for structural proteins like collagen and actin. Transcripts associated with the ‘Complement System’ showed decreased mRNA abundances in group L, highlighting genes of the classical complement pathway. Regarding Bio-functions, the categories ‘Cellular Movement’ and ‘Cell Morphology’ were altered in both comparisons (Additional file 5). For example, ‘Reorganization of Actin Cytoskeleton’ (L vs H: 5 genes; L vs M: 6 genes) and ‘Attachment of Cells’ (L vs H: 6 genes; L vs M: 6 genes) revealed a decreased state of activation in group L. In both comparisons, *CYP24A1*, which encodes for calcitriol 24-hydroxylase, revealed the highest positive fold change (FC; L vs M: + 5.19; L vs H: + 7.69). *TRPM6* revealed a positive fold change of + 1.80 in group L compared to M. There were various genes shared between L vs M and L vs H with negative fold changes, namely *C1S* (L vs M: − 4.36; L vs H: -3.08), *COL15A1* (L vs M: − 3.35; L vs H: -2.73), *ACTA2* (L vs M: − 2.96; L vs H: -2.63), *FN1* (L vs M: − 2.89; L vs H: − 2.45), and *COL1A2* (L vs M: − 2.78; L vs H: − 2.63). There were no pathways enriched for the comparison M vs H.

**Table 1:** Regulated canonical pathways in jejunum

Canonical pathway	Regulation^#^	p-value	Involved Genes
Hepatic Fibrosis/Hepatic Stellate Cell Activation	L<>M	2.24E-14	*ACTA2* (− 2.96); *COL15A1* (− 3.35); *COL1A1* (− 2.60); *COL1A2* (− 2.78); *COL3A1* (− 2.48); *COL4A2* (− 1.46); *COL6A1* (− 1.89); *COL6A3* (− 1.87); *FLT1* (− 1.72); *FN1* (− 2.89); *IGF2* (− 2.12); *IGFBP3* (− 2.02); *KDR* (− 1.89); *MYL9* (− 1.50); *PDGFRA* (− 1.83); *TGFBR2* (− 1.49)
	L<>H	2.09E-08	*ACTA2* (− 2.63); *COL15A1* (− 2.73); *COL1A2* (− 2.25); *COL6A1* (− 1.93); *EDNRB* (− 2.34); *FLT1* (− 1.41); *FN1* (− 2.45); *IGFBP3* (− 1.89); *MMP2* (− 2.20); *MYL9* (− 1.46); *TGFBR2* (− 1.37)
Complement System	L < M	9.08E-08	*C7* (−2.48); *C1QA* (− 1.77); *C1QB* (− 2.03); *C1QC* (− 2.02); *C1R* (− 2.25); *C1S* (− 4.36)
	L < H	3.27E-06	*C1QA* (− 1.71); *C1QB* (− 1.86); *C1QC* (− 1.70); *C1R* (− 1.85); *C1S* (− 3.08)
RhoA Signaling	L < M	7.72E-07	*ABL2* (+ 1.41); *ACTA2* (− 2.96); *ACTC1* (− 2.64); *ARPC1B* (− 1.43); *MYL9* (− 1.50); *PIP5K1A* (− 1.26); *ROCK2* (+ 1.71); *SEPT4* (− 1.57)
	L < H	9.81E-04	*ACTA2* (−2.63); *MYL9* (− 1.46); *NEDD4* (+ 2.11); *PFN2* (− 1.56); *PLXNA1* (− 1.64)
VEGF Signaling	L < M	1.99E-05	*ACTA2* (− 2.96); *ACTC1* (− 2.46); *FLT1* (− 1.72); *KDR* (− 1.89); *PRKCA* (+ 1.35); *ROCK2* (+ 1.71)

# - L < M and L < H = z-score ≤ − 2; L<>M and L<>H = no activity pattern available. *P*-values were calculated using Fisher’s exact test. Fold changes in parentheses indicate positive (L > M or L > H) or negative (L < M or L < H) transcript abundance

In kidney, the comparison between groups H and M revealed no differentially expressed genes. Comparing group L to M (Table 2), the top three pathways ‘Role of NFAT in Regulation of the Immune Response’, ‘CCR5 Signaling in Macrophages’, and ‘PI3K Signaling in B Lymphocytes’ were associated with transcripts involved in the ‘Cellular Immune Response’. In addition, transcripts associated with calcium-dependent pathways (‘Phospholipase C Signaling’, ‘Calcium Signaling’) showed altered mRNA abundances. Considering the Bio-functions (Additional file 5), the categories ‘Small Molecule Biochemistry’ and ‘Molecular Transport’ were affected in both comparisons. Functional analysis predicted an increased state of activation for ‘Transport of Inorganic Cation’ (20 genes), ‘Release of Ca^2+^’ (9 genes), and ‘Cell Survival’ (33 genes). Simultaneously, the functions ‘Morbidity or Mortality’ (67 genes) and ‘Organismal Death’ (65 genes) were predicted to have a decreased state of activation in group L. *SPP1* (osteopontin) ranked among the most up-regulated genes (+ 2.16) in group L compared to M.

**Table 2:** Regulated canonical pathways in kidney

Canonical pathway	Regulation	p-value	Involved Genes
Role of NFAT in Regulation of the Immune Response	L > M	1.08E-05	*AKT3* (+ 1.49); *CALM3* (− 1.25); *CD3D* (+ 1.59); *CD3E* (+ 1.52); *FCGR3B* (+ 1.64); *GNAS* (+ 1.33); *GNG2* (+ 1.43); *LCP2* (+ 1.69); *NFATC2* (+ 1.51); *PLCB3* (− 1.42); *PLCB4* (+ 1.67)
CCR5 Signaling in Macrophages	L<>M	2.33E-05	*CALM3* (− 1.25); *CCR5* (+ 1.76); *CD3D*(+ 1.59); *CD3E* (+ 1.52); *GNAS* (+ 1.33); *GNG2* (+ 1.43); *PRKCB* (+ 1.55)
PI3K Signaling in B Lymphocytes	L > M	3.17E-05	*AKT3* (+ 1.49); *CALM3* (− 1.25); *CAMK2G* (− 1.40); *NFATC2* (+ 1.51); *PLCB3* (− 1.42); *PLCB4* (+ 1.67); *PRKCB* (+ 1.55); *PTPRC* (+ 1.86); *VAV2* (− 1.37)
Phospholipase C Signaling	L > M	4.59E-05	*CALM3* (− 1.25); *CD3D* (+ 1.59); *CD3E* (+ 1.52); *GNAS* (+ 1.33); *GNG2* (+ 1.43); *LCP2* (+ 1.69); *NFATC2* (+ 1.51); *PLA2G6* (− 1.39); *PLCB3* (− 1.42); *PLCB4* (+ 1.67); *PRKCB* (+ 1.55); *RHOJ* (+ 1.56)
Role of Osteoblasts, Osteoclasts and Chondrocytes in Rheumatoid Arthritis	L > M	4.46E-04	*ADAMTS5* (+ 1.64); *AKT3* (+ 1.49); *CALM3* (− 1.25); *CTSK* (+ 1.54); *FZD7* (+ 1.56); *GSN* (+ 1.64); *IGF1* (+ 1.82); *IL33* (+ 1.83); *NFATC2* (+ 1.51); *SPP1* (+ 2.36)
Calcium Signaling	L < M	7.26E-04	*ACTC1* (− 3.24); *CALM3* (− 1.25); *CAMK2G* (− 1.40); *GRIA3* (− 1.38); *LETM1* (− 1.21); *NFATC2* (+ 1.51); *SLC8A1* (− 1.90)
LXR/RXR Activation	L<>H	3.10E-03	*ARG2* (− 1.48); *SERPINF2* (− 3.08); *TF* (− 2.56)
RhoGDI Signaling	L<>H	8.12E-03	*ACTC1* (− 2.88); *CDH2* (− 2.72); *ESR1* (1.87)

# - L < M = z-score ≤ − 2; L > M = z-score ≥ 2; L<>M and L<>H = no activity pattern available. P-values were calculated using Fisher’s exact test. Fold changes in parentheses indicate positive (L > M or L > H) or negative (L < M or L < H) transcript abundance

### Validation of selected transcripts by RT-qPCR

To validate the microarray experiment, transcripts involved in immunoregulatory pathways (*NFATC2*, *SPP1*, *C1QC*, *C7*, *C1R*, *C1S*, and *MMP2*) and in P metabolism (*CYP24A1, CYP27A1*, *PTH1R*, *SLC34A3*, and *VDR*) were analyzed by qPCR. Between microarray and qPCR data, the correlation coefficients were highly significant and ranged between 0.61 and 0.94 (Table 3). The fold changes revealed a reliable dimension. In brief, the qPCR data confirmed the selected microarray results and indicated reproducibility of the microarray analysis. It should be noted that the qPCR analysis of *CYP27A1* exhibited significant alterations between the different groups (L > M; L > H; M > H).

**Table 3:** RT-qPCR results and correlation. Values in parentheses represent Fold Changes (FC)

Tissue	Gene name	Microarray			qPCR#			Correlation##
		H vs L	H vs M	M vs L	H vs L	H vs M	M vs L	
Kidney	*CYP27A1*	H < L (2.11)	H < M (1.45)	M < L (1.46)	H < L*** (2.83)	H < M** (1.60)	M < L** (1.77)	0.94
	*NFATC2*	H < L (1.33)	H > M (1.13)	M < L** (1.51)	H < L* (1.76)	H > M (1.04)	M < L* (1.82)	0.66
	*PTH1R*	H > L (1.14)	H < M (1.09)	M > L (1.26)	H > L (1.03)	H < M (1.20)	M > L (1.24)	0.62
	*SLC34A3*	H > L (1.04)	H < M (1.26)	M > L (1.31)	H < L (1.30)	H < M* (1.40)	M > L (1.07)	0.61
	*SPP1*	H < L (1.48)	H > M (1.46)	M < L*** (2.16)	H < L* (2.02)	H > M (1.19)	M < L** (2.41)	0.92
	*VDR*	H > L*** (1.75)	H < M (1.05)	M > L*** (1.84)	H > L*** (1.75)	H < M (1.08)	M > L*** (1.88)	0.76
Jejunum	*CYP24A1*	H < L*** (7.69)	H < M (1.48)	M < L*** (5.19)	H < L** (222.14)	H < M (2.79)	M < L* (79.59)	0.89
	*C1QC*	H > L** (1.70)	H < M (1.18)	M > L*** (2.02)	H > L** (1.79)	H < M (1.05)	M > L** (1.88)	0.83
	*C1R*	H > L** (1.85)	H < M (1.19)	M > L*** (2.24)	H > L** (2.19)	H < M (1.15)	M > L** (2.52)	0.93
	*C1S*	H > L*** (3.08)	H < M (1.19)	M > L*** (4.36)	H > L** (2.48)	H < M (1.34)	M > L*** (3.33)	0.89
	*C7*	H > L (2.02)	H < M (1.18)	M > L** (2.47)	H > L* (2.64)	H < M (1.14)	M > L** (3.00)	0.94
	*MMP2*	H > L*** (2.20)	H > M (1.18)	M > L** (1.88)	H > L** (4.05)	H > M (1.21)	M > L** (3.35)	0.92

Significance level was set at ***p < 0.001; **p < 0.01, *p < 0.05 and q < 0.2 for results from microarray and qPCR analyses. Fold Changes are stated in parentheses. # - Values were calculated by factorial normalization on RPL32 expression values. ## - Correlation of normalized values was calculated by Spearman; the significance level for correlation was set at *p* < 0.01. Scatterplots of jejunal and renal transcripts are displayed in Additional file 6

## Discussion

Growing piglets face the challenge of maintaining P homeostasis despite increased P requirements for body growth and weight gain. Hence, piglets have to orchestrate numerous regulatory mechanisms in different tissues, which is reflected on the endocrine [12] and transcriptional levels.

### Decreased P intake provokes a transcriptional response in the jejunum, but not in the colon

The expression profiles of jejunum and colon resulting from lowered P intake exhibited considerable differences in the tissue-specific regulation of transcription. In jejunum, numerous transcripts were differentially expressed between the dietary groups, whereas the colon showed barely any transcriptional differences, revealing a variable contribution to maintain P homoeostasis in specific parts of the intestine. The jejunum is assumed to be the major site for active P absorption in monogastric mammals [28, 29]. P absorption is hormonally stimulated by calcitriol [30], whose serum levels have been demonstrated previously to be increased in the group L [12]. In fact, calcitriol is able to induce the expression of target genes involved in enteral absorption, renal excretion and bone remodeling which affect calcium absorption and renal P excretion [31]. In jejunum, *CYP24A1* exhibited the highest positive fold change in group L compared to groups H and M. In fact, *CYP24A1* transcription is known to be strongly regulated by calcitriol as the encoded 24-hydroxylase inactivates calcitriol. This also corresponds to our observations in a pig experiment in which the jejunal transcription of *CYP24A1* varied greatly in diets with variable calcium-P ratios [32]. This feedback loop could prevent hypercalcemic effects in L-samples, but could also have effects on P-absorption in the jejunum [33]. In addition, there are a number of genes encoding for proteins responsible for P uptake in jejunum. From these genes, *SLC34A2*, encoding the sodium dependent phosphate cotransporter type IIb, and *S100G*, encoding calbindin-D9k, showed no mRNA alterations in our study. These findings are supported by Saddoris et al., who assumed a post-transcriptional regulation of *SLC34A2* and *S100G* [11].

The jejunum represents an important site for absorption of other minerals. Our results revealed that the expression of the transient receptor potential cation channel subfamily M member 6 (*TRPM6*) was diet-dependently altered (L > M). In fact, TRPM6 is essential for magnesium absorption [34]. As previously presented by our group [12], the analyzed pigs exhibited diet-dependent magnesium levels in serum (L < M; H < M). This observation supports previous studies stating an interrelation between magnesium, calcium, and inorganic P [35, 36]. However, mRNA expression of *TRPM6* was increased in L but not in H animals when compared to M fed animals. This pattern might reflect compensatory attempts of L animals to maintain mineral homoeostasis.

Our results indicate that the large intestine has no part in P absorption and that the differentially expressed transcripts in colon are not associated with mineral homeostasis or intestinal functions. As reviewed by Sabbagh et al., P absorption is physiologically irrelevant in the colon under normal conditions; in fact, Breves and Schröder could not detect P absorption in the large intestine of pigs [28, 37]. Therefore, our microarray experiment in the colon validated previous results and supports the finding that the transcriptional response in jejunum is specific to P diets.

### Transcriptional response in kidney to lowered P intake reveals the importance of *CASR* for mineral homeostasis

**Fig. 1:**
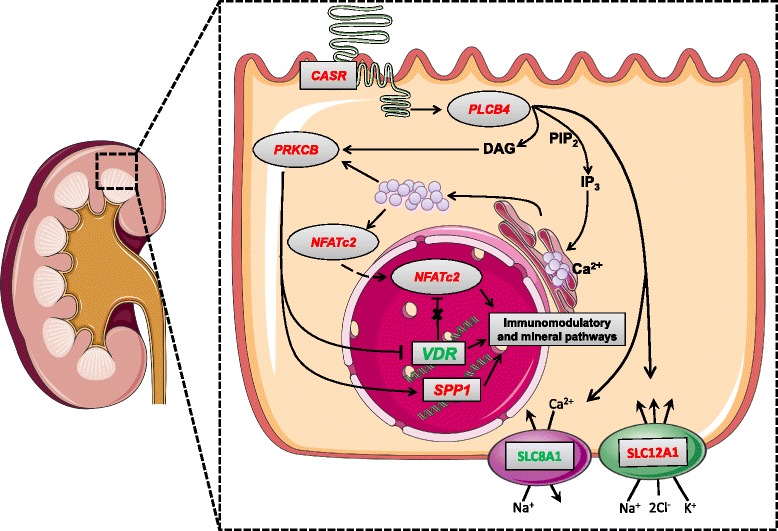
Model for signal transduction in renal cells due to lowered P intake. Solid arrows represent activation, dashed arrows represent inhibition. The data is based on a microarray comparison between groups L and M. Genes in red: L > M; genes in green: L < M. L – Low P supply; M – Medium P supply; H – High P supply

Of the three investigated organs, the kidney exhibited the highest number of differentially expressed genes. The supporting qPCR analysis revealed that the mRNA abundance of *CYP27A1* was increased in pigs with lowered P intake (L > M; L > H; M > H). The enzyme (CYP27A1) has 25-hydroxylase activity to convert cholecalciferol to calcidiol [38]. Calcidiol is the precursor of calcitriol and acts as a transport and storage form of vitamin D [39]. Since *CYP27A1* is known to be primarily expressed in liver tissue, the diet-dependent *CYP27A1* expression in kidney reflects that mineral homoeostasis comprises feedback mechanisms at local tissue sites. Apart from this known physiological mechanism, there were several canonical pathways associated with calcium-dependent signaling that were altered by P diet. In particular, the phospholipase C beta 4 (*PLCB4*; L > M) was significantly altered by P intake, implicating a change in signal transduction. The catabolic activity of phospholipase C depends on the activation of G protein-coupled receptors [40] such as the calcium sensing receptor (*CASR*). Consequently, *CASR* mRNA abundance was increased in group L (Fig. 1). In fact, *CASR* expression was probably induced by the elevated levels of calcium in serum observed in group L [12]. It is known from kidney that CASR is involved in the control of calcium and P homeostasis, transport of cations, urinary acidification and concentration, and renin release [41]. Activated PLCB4 catalyzes the conversion of phosphatidylinositol 4,5-bisphosphate (PIP2) to inositol 1,4,5-trisphosphate (IP3) and diacylglycerol (DAG). IP3 and DAG are both essential as second messengers for numerous cellular and metabolic processes [42]. Functional analysis predicted an increased state of activation for ‘release of Ca^2+^’, which is consistent with the fact that IP3 induces the release of calcium from intracellular stores into the cytoplasm, thus activating downstream signaling cascades [43]. DAG and calcium are necessary to activate protein kinase C beta (*PRKCB*), which in turn has various functions in diverse cellular signaling pathways [44]. Our results showed increased mRNA abundance for *PRKCB* and decreased transcript levels for *VDR*. This is consistent with findings demonstrating that the elevation of intracellular calcium and activation of protein kinase C inhibits vitamin D receptor (*VDR*) gene expression [45]. The activation of protein kinase C has been shown to enhance the induction of *SPP1* by calcitriol but is not correlated with *VDR* activation [46]. As reviewed elsewhere [47], osteopontin, encoded by *SPP1*, has various functions in different cell types. In kidney, the protein is synthesized in the epithelial cells and secreted into urine. There, osteopontin inhibits the urinary crystallization of calcium salts, thus avoiding the formation of renal stones and increasing urinary calcium excretion [48]. Moreover, our results showed increased mRNA abundance of *STC1* in group L. *STC1* encodes stanniocalcin, which enhances P re−/absorption in small intestine and kidney [49]. Circulating calcium and calcitriol [12] induce *STC1* gene expression [50], whereby these effects are possibly mediated by activation of CASR [51].

Our results also reveal a number of P-regulated genes encoding transporter molecules. The kidney-specific proteins NKCC2, encoded by *SLC12A1* (L > M), and NCC, encoded by *SLC12A3* (L < M), are responsible for renal sodium chloride absorption [52]. Another target transport protein is NCX1, encoded by *SLC8A1* (L < M), which regulates intracellular calcium homeostasis [53]. Furthermore, the phospholipases *PLCB3* (L < M) and *PLA2G6* (L < M) modulate electrolyte transport by CASR signaling [41, 54, 55]. The proteins NPT4, encoded by *SLC17A3* (L < M), and NPT5, encoded by *SLC17A4* (L < M), mediate the transport of organic anions such as urate. Unfortunately, there is not much known about their regulation and functional physiology [56]. However, our results suggest that changes in serum P and calcium levels are associated with altered mRNA of various transporter proteins in kidney in order to maintain mineral homeostasis.

### Genes involved in immune-relevant pathways are altered in jejunum by decreased P intake

The gene expression in jejunum revealed considerable shifts due to low P intake, resulting primarily in the downregulation of genes involved in several signaling pathways. The top canonical pathway ‘Hepatic fibrosis’ might state an accumulation of extracellular matrix proteins (mainly collagen) which can occur in all tissues. The pathways ‘RhoA Signaling’ and ‘VEGF Signaling’ comprise genes associated with cytoskeleton organization like actin, myosin, and Rho Kinase 2. Interestingly, Suyama et al. demonstrated an upregulation for collagen fibril organization in rat kidney due to a high P diet, confirmed by fibrosis-like regions in kidney sections [57]. During fibrogenesis, the intestinal function is restricted due to impaired motility and deposition of collagens [58]. Additionally, it is known from other diseases that deposition of collagen can impair the immune function of GALT [59]. Furthermore, Kutuzova and DeLuca demonstrated that calcitriol suppresses various matrix modeling proteins which increases the intestinal epithelial tight junction permeability [60]. Consequently, the downregulation of various genes associated with the extracellular matrix (e.g. *COL1A1*, *COL1A2*, *SPARC*, and *MMP2*) and cytoskeleton (*ACTC1*, *ACTA2*, *MYL9*, and *KDR*) indicates that a low P intake has not only anti-calcific features and counteracts fibrous changes in the small intestine, but also regulates paracellular calcium absorption through calcitriol actions. Apart from this, jejunal transcripts associated with the ‘Complement System’ showed lowered mRNA abundances due to the low P diet. The complement system acts as an immune surveillance system, discriminating between healthy tissue, apoptotic cells, and microbial intruders [61]. The C1 complex, encoded by *C1QA*, *C1QB*, and *C1QC*, recognizes surface structures on microbial and apoptotic cells and activates the proteases C1r and C1s, initiating the classical pathway of the complement system. All genes, which encode for the C1 complex, were downregulated in group L, indicating an inhibition of the classical pathway. It can be argued that such an inhibition could prevent the immune response to commensal microorganisms, which can be considered as an increase in immune tolerance by blocking the classical pathway and saving energy resources [62]. Taken together, our results indicate that a low P diet has an effect on immune features in jejunum and possibly supports a healthy intestinal function through calcitriol actions.

### Low P results in transcriptional changes involved in mineral homeostasis and immunomodulatory signaling pathways

In kidney, the top three P-regulated pathways were found to be associated with transcripts involved in the cellular immune response. The canonical pathways ‘Role of NFAT in Regulation of the Immune Response’ and ‘Phospholipase C Signaling’ are partly associated with the same transcripts, indicating that they are integrated. Of particular interest is the nuclear factor of activated T cells 2 (*NFATC2*; L > M), a transcription factor that integrates calcium signaling with other signaling pathways and regulates different aspects of the immune system. In fact, the previously described CASR mediated pathway is able to induce the activation of NFATC2 [63]. Our results exhibited increased mRNA abundance for *CD3D*, *CD3E*, and *PTPRC*, encoding for the CD3 complex and CD45, respectively. The CD3 complex is part of the T cell receptor and CD45 is required for T cell activation, both implicating altered T cell signaling as a result of variable dietary P. Activation of the T cell receptor and subsequent involvement of NFAT proteins leads to T helper cell differentiation and induction of immune genes [64]. Furthermore, an unbalanced activation of NFAT without co-stimulatory molecules (e.g. CD28) leads to the expression of anergy-inducing genes, mediating a status of T cell unresponsiveness [65, 66]. Moreover, NFATC2 is also a molecular target for calcitriol-dependent effects mediated by VDR, which is able to inhibit the NFATC2 complex formation via AP-1 (activator protein 1) [67, 68]. Thus, the potential down-regulation of *VDR* by protein kinase C might enable the formation of the NFATC2 complex. Calcitriol has immunoregulatory properties, such as T cell activation [69] as well as T cell suppression [67]. These contrary regulatory effects on the immune system of vitamin D are mediated by VDR expression level [70], which in turn is modulated by the activity of protein kinase C. Also of interest is osteopontin, which is not only an inhibitor of urinary crystallization, but has also immunomodulatory properties like T cell activation and leucocyte recruitment [47]. The functions and mechanisms of osteopontin are diverse, and its expression is affected by various factors, including calcitriol, calcium and P. Thus, osteopontin might function as a further link between mineral homeostasis and the immune system, but the distinct molecular routes due to modulated P supply remain unclear. The genes *NFATC2*, *VDR*, and *SPP1* are targets of pathways sensitive to mineral alterations as well as T cell receptor signaling, highlighting the interaction between P and the immune system.

## Conclusion

We characterized the transcriptional response in re−/absorbing and excreting tissues in pigs after variations in dietary P intake. Our analyses in jejunum, colon, and kidney revealed changes in the expression of various genes involved in adaptive responses to maintaining mineral homeostasis. The altered serum calcium, PTH and calcitriol levels observed previously [12] may trigger cellular signaling responses involving genes identified by this study. Specifically, transcripts associated with the complement system and NFAT signaling are likely to represent the involvement of P in the immune system. Furthermore, the P-dependent expression of osteopontin might link immune modulation and mineral homeostasis. However, the influence of a varying P intake during an immune challenge remains to be investigated. Taken together, our results suggest that the impact of reduced P supply during the growth period of piglets should be considered in terms of immune function, growth and performance. Possible phased feeding regimes covering age-related mineral requirements should therefore be studied in order to reconcile aspects of livestock production and animal welfare in order to derive a relevant P policy/governance.

## Additional files

Additional file 1 Composition of the experimental diets (XLSX 11 kb)

Additional file 2 Primer used for verification of microarray by qRT-PCR (XLSX 11 kb)

Additional file 3 Probe-sets showing significantly altered mRNA abundances (per diet and tissue). The Venn diagram depicting the number of probe-sets differentially expressed in a) colon, b) jejunum, and c) kidney. Number of up-regulated probe-sets is shown in red. Number of down-regulated probe-sets is shown in green. L – Low P supply; M – Medium P supply; H – High P supply (XLSX 254 kb)

Additional file 4 The hierarchical clustering of filtered probe-sets is represented by log2 transformed intensity values obtained (i) from the variance component diet*tissue (least square means) and (ii) from data obtained from individual animals. L – Low P supply; M – Medium P supply; H – High P supply (PDF 2172 kb)

Additional file 5 Molecular and cellular functions affected by P supply (XLSX 9 kb)

Additional file 6 Scatterplots of jejunal and renal transcripts (PDF 20 kb)

## Abbreviations

ATP: Adenosine triphosphate
Ca: Calcium
FC: Fold change
GALT: Gut-associated lymphoid tissue
H: Group high
IPA: Ingenuity Pathway Analysis
L: Group low
M: Group medium
P: Phosphorus
$$ {\mathrm{PO}}_4^{3-} $$: Phospate
qPCR: Real-time quantitative PCR
RMA: Robust Multiarray Average
SD: Standard deviation
x̅: Mean
