# Supplementary material for: Lowered dietary phosphorus affects intestinal and renal gene expression to maintain mineral homeostasis with immunomodulatory implications in weaned piglets
Source: BMC Genomics. 2018 Mar 20;19:207. doi: 10.1186/s12864-018-4584-2 (PMC5859397; doi:10.1186/s12864-018-4584-2)
Supplement: Supplementary file 4 — The hierarchical clustering of filtered probe-sets is represented by log2 transformed intensity values obtained (i) from the variance component diet*tissue (least square means) and (ii) from data obtained from individual animals. L – Low P supply; M – Medium P supply; H – High P supply (PDF 2172 kb) [file 12864_2018_4584_MOESM4_ESM.pdf]

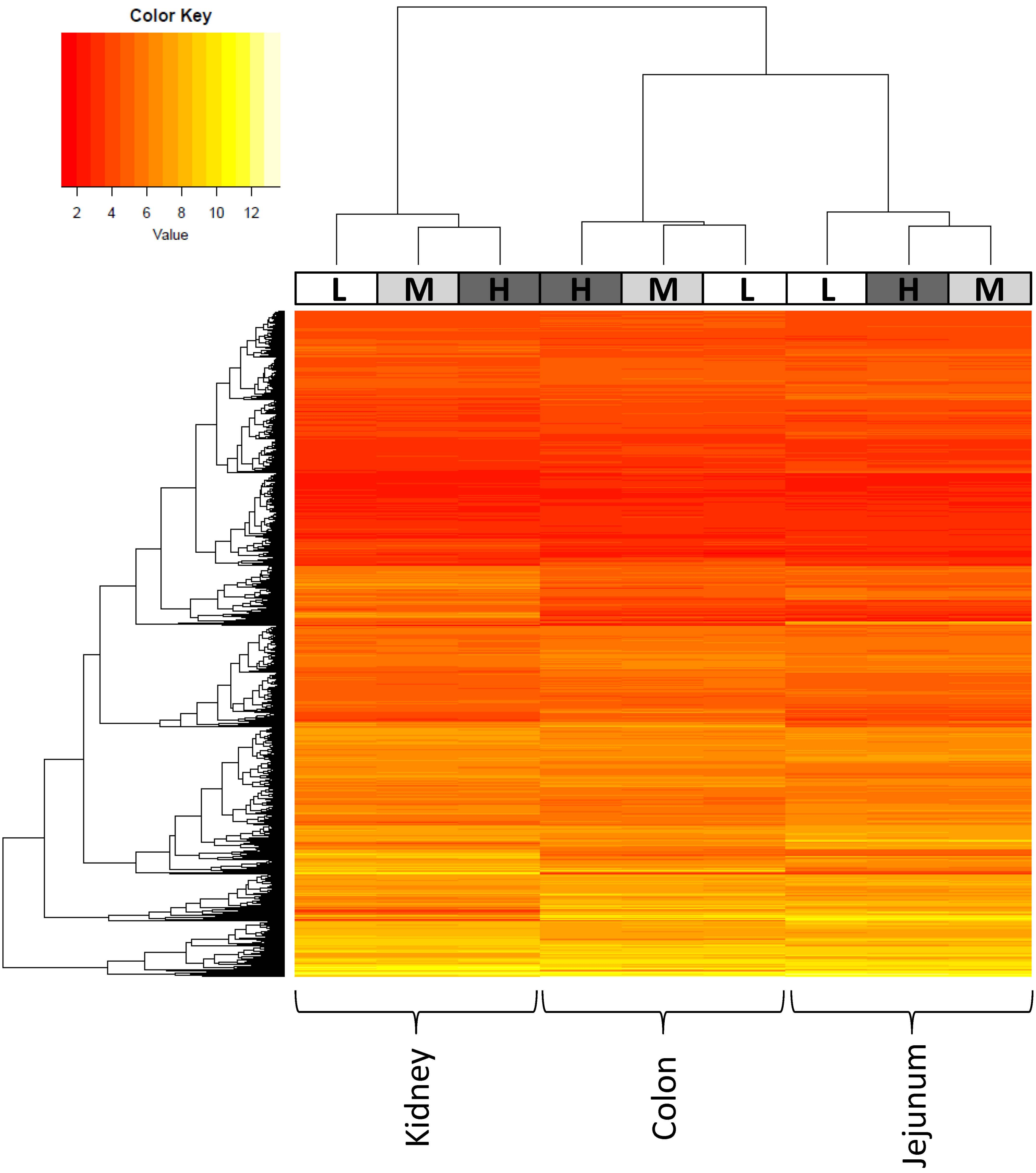

Color Key
2
4
6
8
10
12
Value
L
M
H
H
M
L
L
H
M
Kidney
Colon
Jejunum

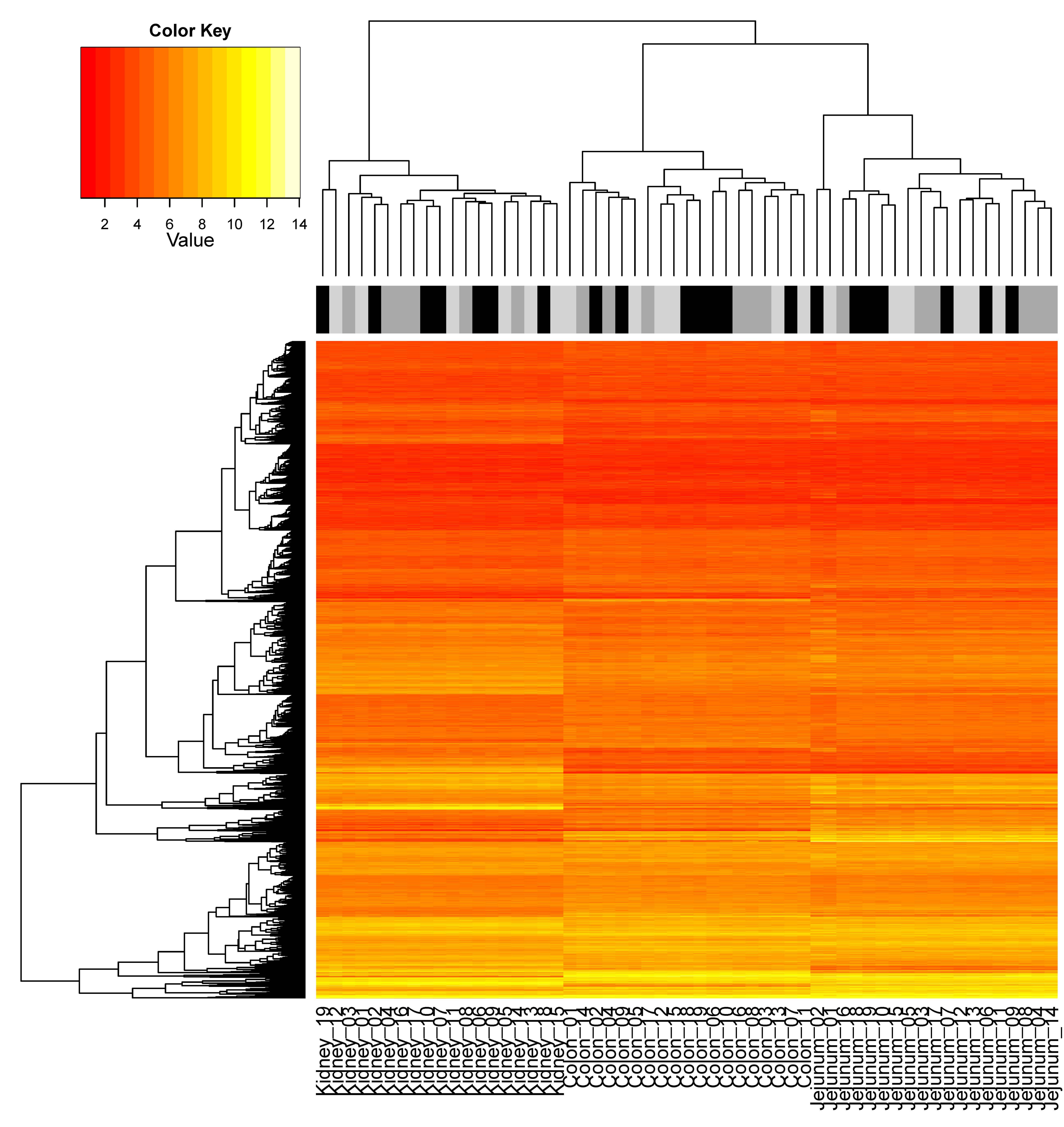
Color Key
2 4 6 8 10 12 14
Value
