# Supplementary figures and images for: Lowered dietary phosphorus affects intestinal and renal gene expression to maintain mineral homeostasis with immunomodulatory implications in weaned piglets
Source: BMC Genomics. 2018 Mar 20;19:207. doi: 10.1186/s12864-018-4584-2 (PMC5859397; doi:10.1186/s12864-018-4584-2)

CYP27A1

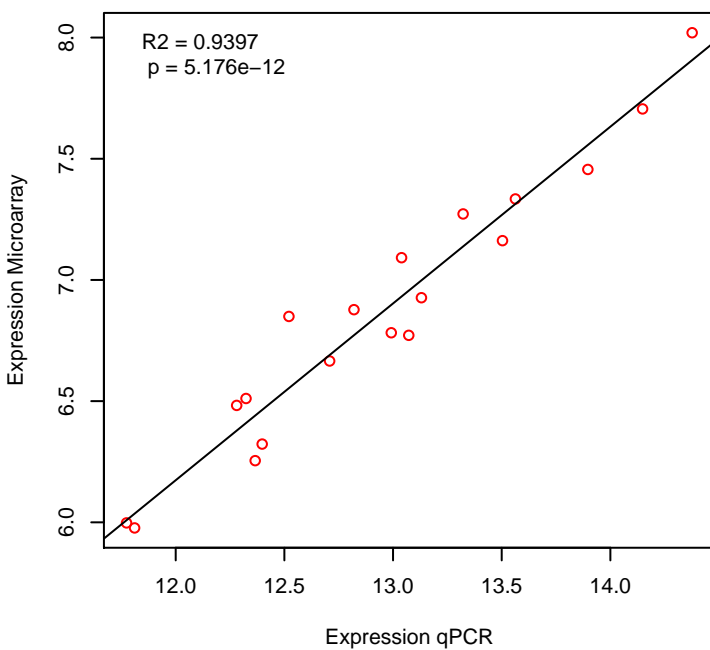

NFATC2

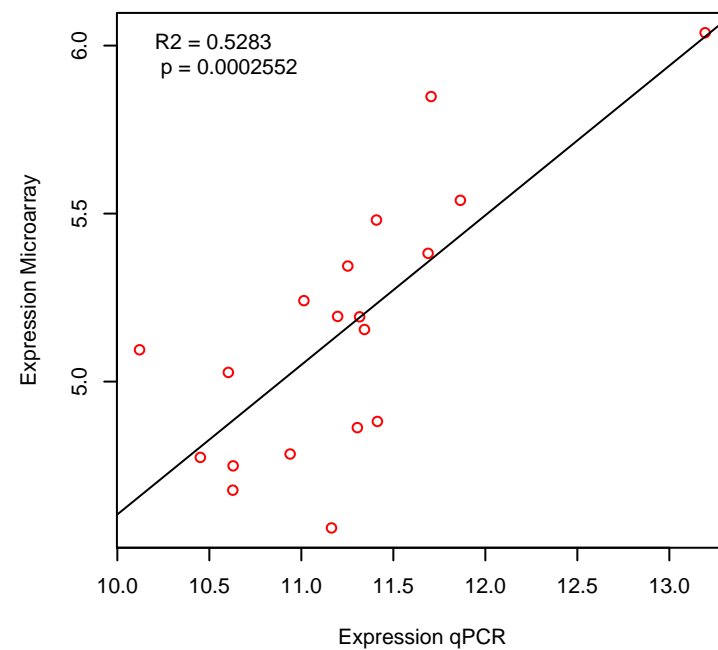

PTH1R

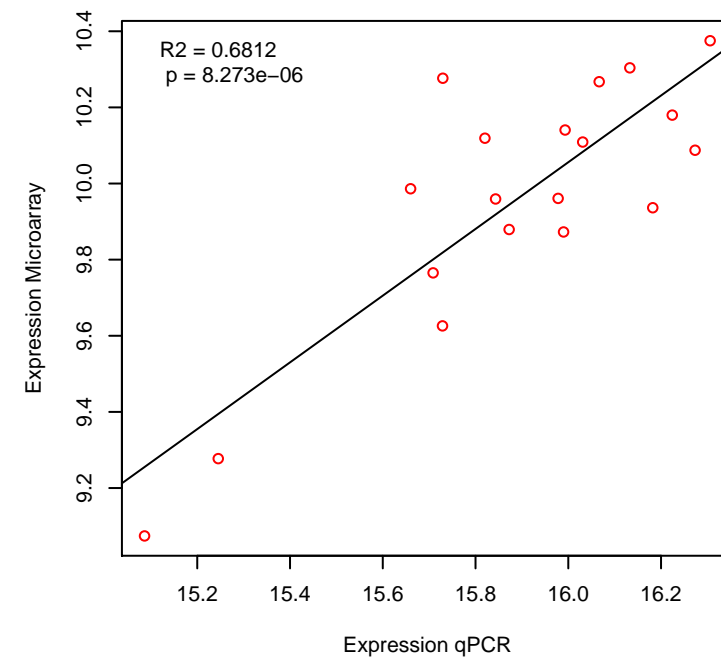

SLC34A3

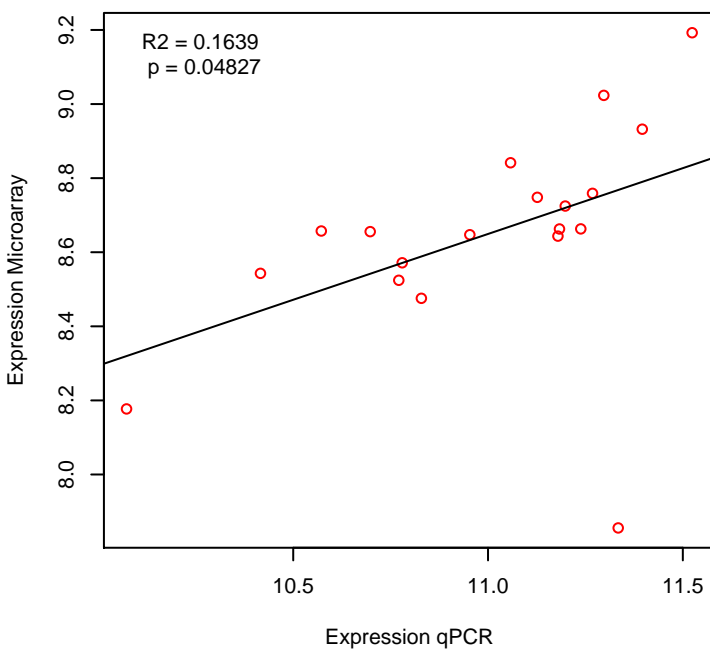

SPP1

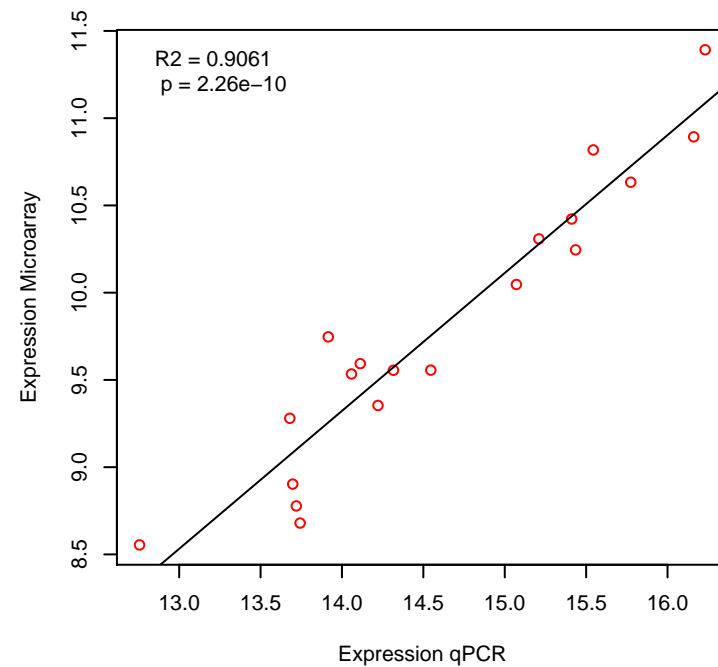

VDR

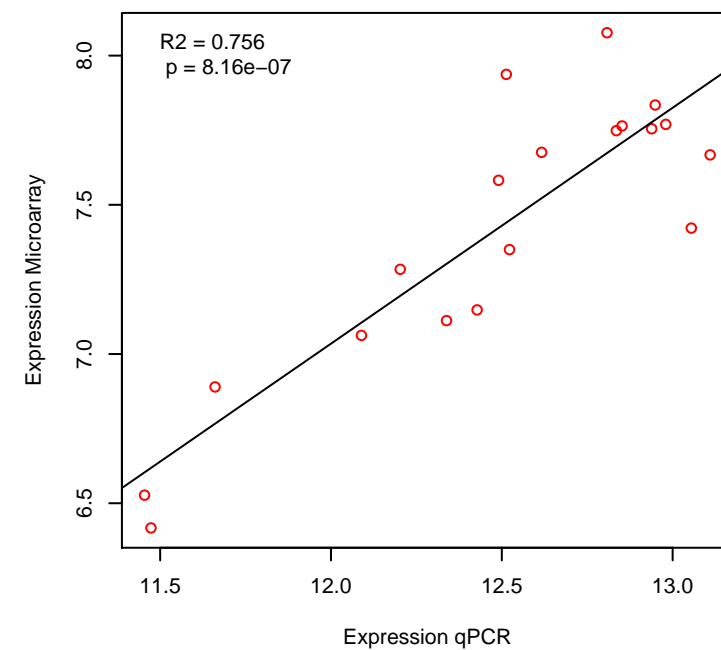

CYP24A1

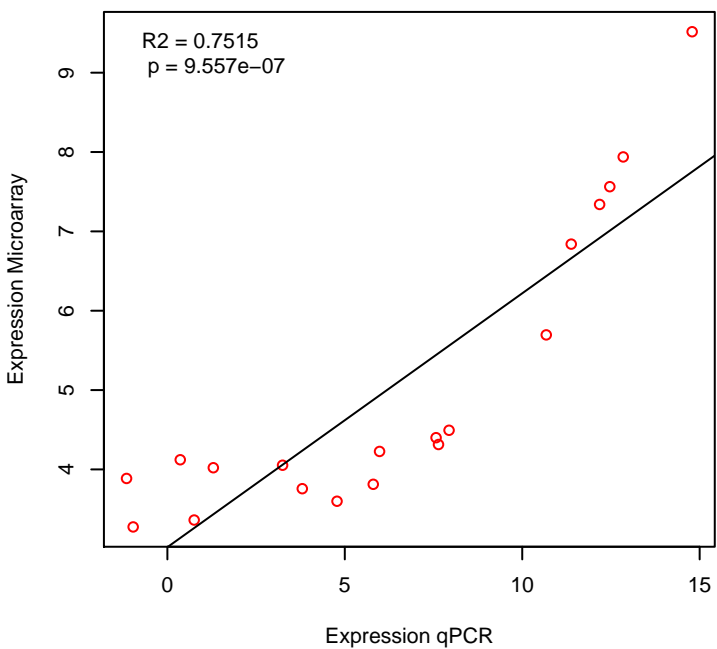

C1QC

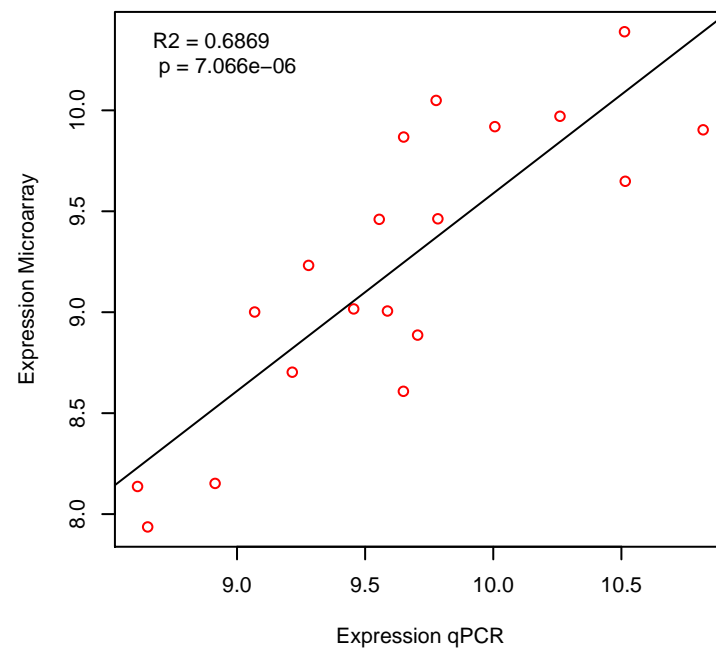

C1R

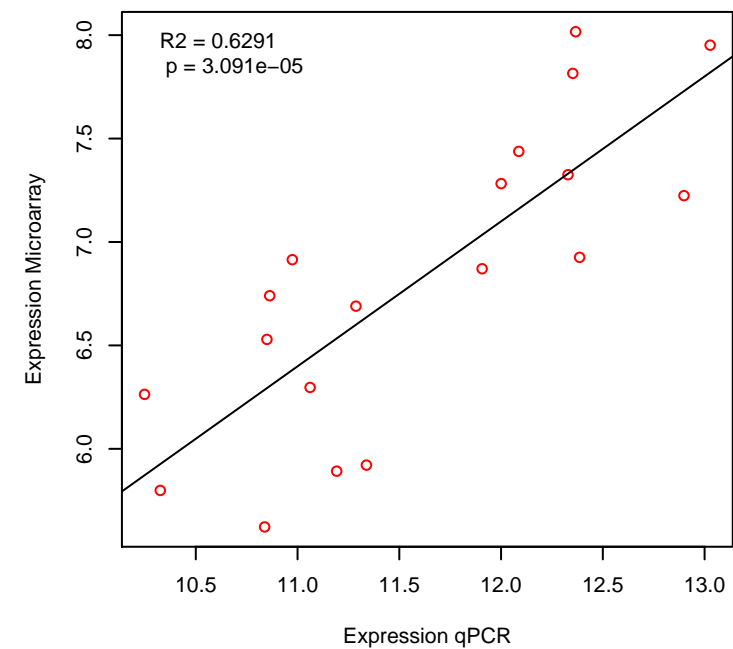

C1S

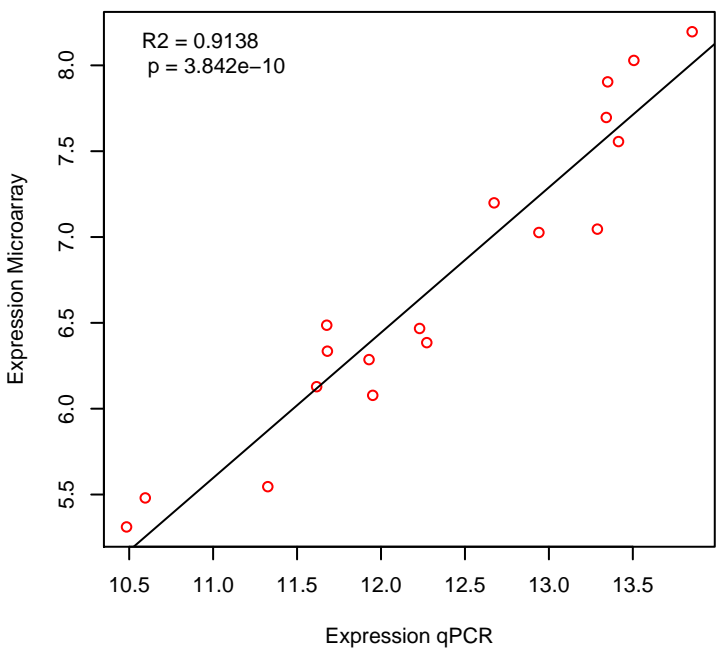

C7

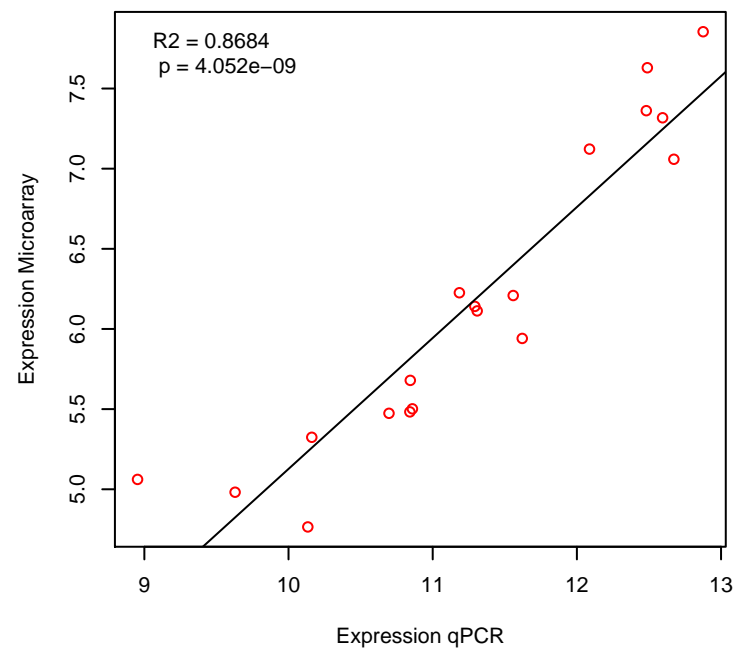

MMP2

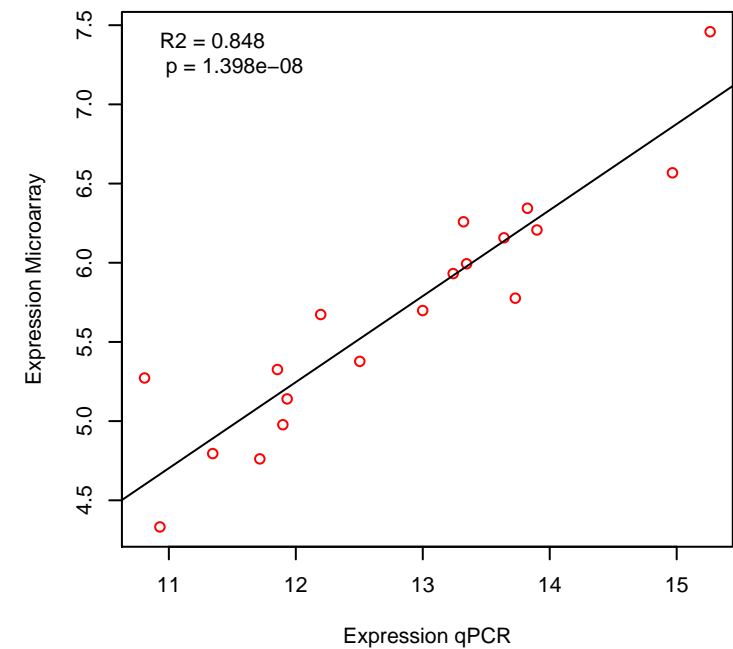

Supplement: Supplementary file 6 — Scatterplots of jejunal and renal transcripts (PDF 20 kb) [file 12864_2018_4584_MOESM6_ESM.pdf]
